# An open label trial of folate receptor-targeted intraoperative molecular imaging to localize pulmonary squamous cell carcinomas

**DOI:** 10.18632/oncotarget.24399

**Published:** 2018-02-05

**Authors:** Jarrod D. Predina, Andrew D. Newton, Leilei Xia, Christopher Corbett, Courtney Connolly, Michael Shin, Lydia Frezel Sulyok, Leslie Litzky, Charuhas Deshpande, Shuming Nie, Sumith A. Kularatne, Philip S. Low, Sunil Singhal

**Affiliations:** ^1^ Center for Precision Surgery, Perelman School of Medicine at The University of Pennsylvania, Philadelphia, PA, USA; ^2^ Division of Thoracic Surgery, Department of Surgery, Perelman School of Medicine at The University of Pennsylvania, Philadelphia, PA, USA; ^3^ Department of Surgery, Perelman School of Medicine at The University of Pennsylvania, Philadelphia, PA, USA; ^4^ Division of Urology, Department of Surgery, Perelman School of Medicine at The University of Pennsylvania, Philadelphia, PA, USA; ^5^ Pathology and Laboratory Medicine at The Hospital of The University of Pennsylvania, Philadelphia, PA, USA; ^6^ Department Biomedical Engineering and Winship Cancer Institute, Emory University, Atlanta, GA, USA; ^7^ Department of Chemistry, and Purdue Institute for Drug Discovery, Purdue University, West Lafayette, IN, USA

**Keywords:** squamous cell carcinoma, surgery, molecular imaging, folate receptor

## Abstract

**Background:**

Clinical applicability of folate receptor-targeted intraoperative molecular imaging (FR-IMI) has been established for surgically resectable pulmonary adenocarcinoma. A role for FR-IMI in other lung cancer histologies has not been studied. In this study, we evaluate feasibility of FR-IMI in patients undergoing pulmonary resection for squamous cell carcinomas (SCCs).

**Methods:**

In a human clinical trial (NCT02602119), twelve subjects with pulmonary SCCs underwent FR-IMI with a near-infrared contrast agent that targets the folate receptor-α (FRα), OTL38. Near-infrared signal from tumors and benign lung was quantified to calculate tumor-to-background ratios (TBR). Folate receptor-alpha expression was characterized, and histopathologic correlative analyses were performed to evaluate patterns of OTL38 accumulation. An exploratory analysis was performed to determine patient and histopathologic variables that predict tumor fluorescence.

**Results:**

9 of 13 SCCs (in 9 of 12 of subjects) displayed intraoperative fluorescence upon NIR evaluation (median TBR, 3.9). OTL38 accumulated within SCCs in a FRα-dependent manner. FR-IMI was reliable in localizing nodules as small as 1.1 cm, and prevented conversion to thoracotomy for nodule localization in three subjects. Upon evaluation of patient and histopathologic variables, *in situ* fluorescence was associated with distance from the pleural surface, and was independent of alternative variables including tumor size and metabolic activity.

**Conclusions:**

This work demonstrates that FR-IMI is potentially feasible in 70% of SCC patients, and that molecular imaging can improve localization during minimally invasive pulmonary resection. These findings complement previous data demonstrating that ∼98% of pulmonary adenocarcinomas are localized during FR-IMI and suggest broad applicability for NSCLC patients undergoing resection.

## INTRODUCTION

Each year, nearly 200,000 Americans are diagnosed with non-small cell lung cancer (NSCLC) [1]. Surgical resection provides the best opportunity for long-term survivorship for NSCLC patients, and nearly 80,000 patients undergo pulmonary resection in the United States annually [1]. Although the use of minimally invasive pulmonary resection (video-assisted thoracic surgery or robotic surgery) for NSCLC is common, these approaches limit a surgeon’s ability to visually inspect and palpate the entire lung [2]. Consequential incomplete intraoperative information during minimally invasive pulmonary resection can make localization and adequate resection of tumors challenging.

Intraoperative molecular imaging (IMI), also known as fluorescence guided surgery, is a rapidly evolving technique that has been demonstrated to improve the surgeon’s ability to accurately identify malignant nodules during oncologic resection [3–10]. This approach incorporates the systemic delivery of optical contrast agents which preferentially accumulate in malignant tissues, and allows for real-time fluorescent imaging using calibrated camera systems. IMI has been effectively utilized for a variety of malignancies including thoracic malignancies [11–14], central nervous system tumors [15], head and neck cancers [8], ovarian carcinomas [5], and thymoma [16].

Over the last decade our group has initiated several human clinical trials involving folate receptor-targeted intraoperative molecular imaging (FR-IMI) for the most common NSCLC histology, pulmonary adenocarcinomas [3, 11, 13, 17–19]. Pulmonary adenocarcinomas were chosen as an initial disease histology given a high level of FRα expression which approaches 85% [13, 20, 21]. In a Phase I trial, we found FR-IMI with OTL38 to be both safe and feasible. After optimizing imaging parameters, we appreciated that more than 90% of pulmonary adenocarcinomas accumulated OTL38 and displayed fluorescence *in situ* or *ex vivo* [13]. In a follow-up study involving 50 subjects, the addition of FR-IMI to standard of care intraoperative assessment allowed for detection of occult subcentimeter nodules in 12% of subjects [3]. Together, these intraoperative findings impacted operative planning, tumor staging, and postoperative adjuvant therapy treatment.

Although initial trials with FR-IMI have been successful for localization of pulmonary adenocarcinomas, utility for other NSCLC histologies remains undefined. Pulmonary squamous cell carcinomas (SCCs) account for approximately 30% of all lung cancers and 35% of NSCLCs, thus exploration of IMI with OTL38 in SCCs has the potential for high clinical impact [22]. Unfortunately, there is currently a paucity of information describing FRα expression patterns in SCCs [20, 21]. Further, the few reports describing FRα expression provide conflicting data, with FRα expression levels ranging from 10% to 60% [20, 21]. This limited information has impeded progress of folate-receptor targeted molecular imaging for SCCs.

As our group prepares to move FR-IMI with OTL38 to Phase II evaluations, we sought to better understand the utility of FR-IMI in patients with SCC. In this report, we evaluate FR-IMI with OTL38 in 12 subjects undergoing minimally invasive pulmonary resection for SCCs. In this study, our primary goals were to determine if OTL38 reliably accumulates within SCC and generates tumor fluorescence. Our secondary goals were to characterize folate receptor expression within SCC's and determine factors which contribute to tumor fluorescence during FR-IMI.

## RESULTS

### Subject and drug delivery data

Between August 2015 and February 2017, 12 subjects with a histologically confirmed diagnosis of pulmonary SCC underwent VATS resection with FR-IMI with OTL38. Seven included subjects were male and 5 were female. The median age of subjects at resection was 70 years (IQR, 68-77 years). Except for Subject 5, all subjects had a solitary pulmonary nodule by preoperative imaging. Subject 5 had presented with two synchronous nodules: one in the left upper lobe and one in the left lower lobe. Preoperative CT and PET were obtained in all subjects and the median tumor size of SCCs included in analysis was 1.7 cm (IQR, 1.5-4.0 cm) with median SUV of 4.8 (IQR, 3.5-8.9). A full summary of patient characteristics is provided in Table 1.

**Table 1:** Subject characteristics

ID	Age (years)	Gender	Tumor Location	Size (cm)	Depth (cm)	SUV	*In situ* TBR	*Ex vivo TBR*	Impact of IMI
1	70	F	RLL	5.2	2.0	13.1	1.3	4.3	
2	87	F	RUL	1.0	0.1	avid^*^	1.6	1.8	
3	77	F	LUL	1.7	0.0	8.7	3.7	4.2	
4^**^	60	F	LUL LLL	0.9 1.2	0.0 0.4	3.0 3.5	1.0 1.2	1.9 1.9	
5	69	M	RLL	1.5	0.5	8.9	2.2	5.6	Improved minimally invasive localization
6	77	M	RLL	4.6	0.2	8	2.4	2.6	
7	70	M	RUL	4.0	0.0	18.3	3.8	3.9	
8	67	M	RLL	2.8	0.0	5.1	3.0	3.8	
9	78	M	RUL	1.1	1.2	3.1	2.2	3.5	Improved minimally invasive localization
10	72	M	RUL	3.0	4.0	4.5	1.0	3.9	
11	66	M	RUL	4.5	2.5	2.5	1.3	1.4	
12	70	F	RUL	1.7	0.9	3.8	2.1	2.4	Improved minimally invasive localization

M-Male; F-Female; RUL-Right Upper Lobe; RML-Right Middle Low; RLL-Right Lower Lobe; LUL-Left Upper Lobe; LLL-Left Lower Lobe.

^*^ unable to quantify as records from outside our health system.

^**^ Subject with two nodules identified preoperatively. The larger was PET avid, with suggesting SCC. The second nodule was not biopsied preoperatively and was not PET avid; however, proved to be SCC on final histopathologic analysis.

Subjects received a median of 2.07 mg (IQR, 1.77-2.58 mg) of OTL38 4.4 hours (IQR, 3.4-5.1 hours) prior to resection and imaging (Table 2). Each subject was administered the full amount of study drug. No adverse events were noted during 30 days of follow-up.

**Table 2:** Toxicology data

ID	Weight (kg)	OTL38 given (mg)	Adverse Event^*^	Time from Infusion to Imaging (hours)
1	61.8	1.55		2.97
2	63.5	1.59		3.38
3	61.7	1.54		5.73
4	79.8	2.00		4.40
5	134.3	3.36		4.72
6	102.1	2.55		6.5
7	107	2.68		6.45
8	73.5	1.84		3.68
9	82.8	2.07		5.05
10	103	2.58		3.15
11	70.8	1.77		4.18
12	88.5	2.21		2.98

^*^Adverse Events were graded using the Common Terminology Criteria for Adverse Events (Version 4.03) [33].

### SCCs displaying fluorescence during FR-IMI

**Figure 1:**
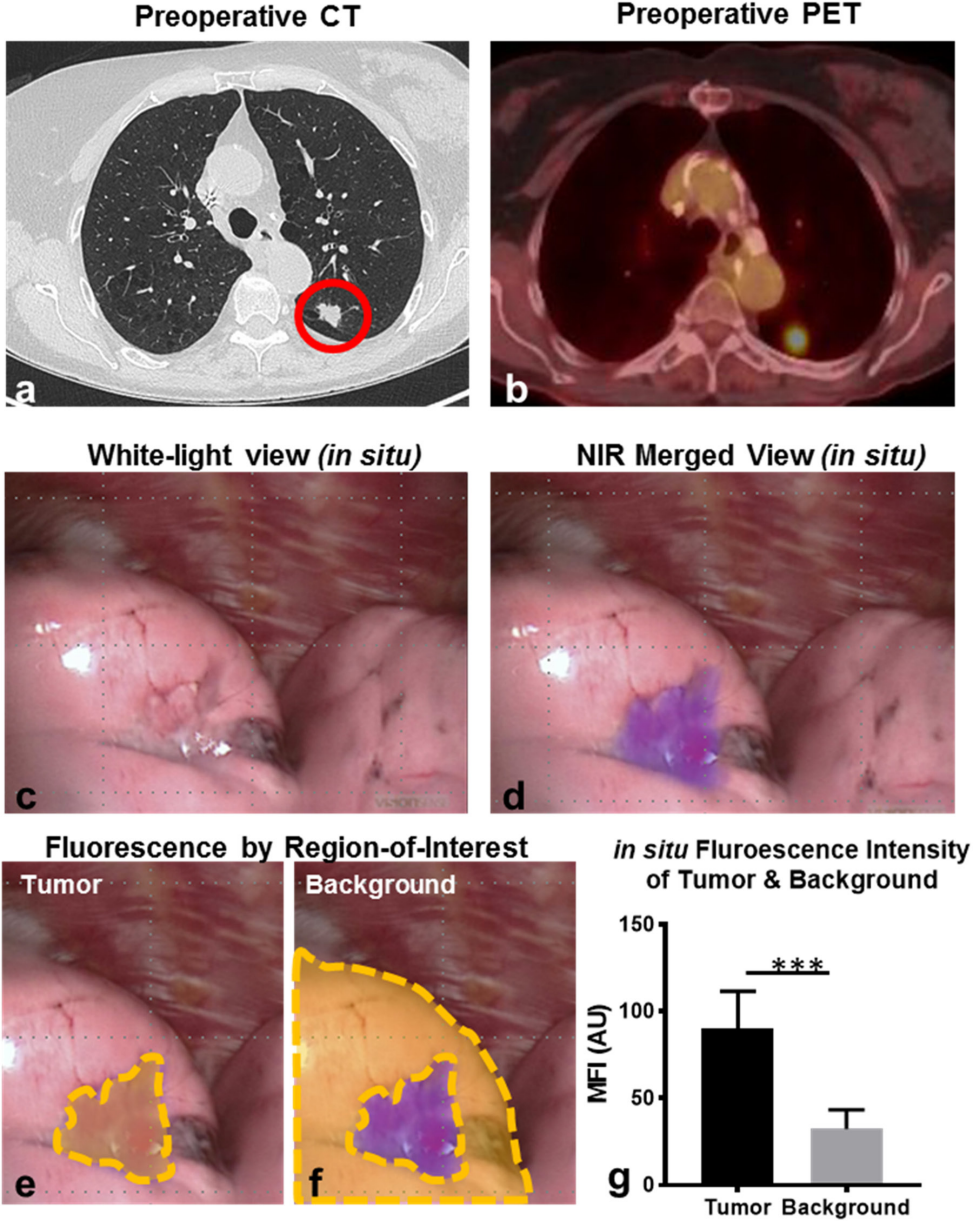
Pulmonary SCCs display *in situ* fluorescence during FR-IMI with OTL38 Representative example: Subject 3 presented with a 1.7cm left upper lobe nodule by preoperative CT **(a)**. Preoperative PET displayed an SUV of 8.7 **(b)**. During FR-IMI the nodule was localized during standard thoracoscopic views **(c)**, and displayed strong NIR signal during fluorescent imaging **(d)**. Median fluorescent intensity (MFI) was determined for ROIs corresponding to tumor **(e)** and benign lung (background) **(f)**. MFIs for ROIs were compared for SCCs (n=7) displaying *in situ* signal **(g)**. Red circle-pulmonary SCC; yellow gates-ROIs measured by fluorescent analysis; ^***^p<0.001.

*In situ* molecular imaging using the Iridium’s thoracoscopic configuration identified tumor-specific fluorescence signal through the pleural surface in 7 (58.3%) of 12 subjects (Figure 1a-1d; Supplementary Video 1), with 7 of 13 (53.8%) nodules being localized by NIR imaging. Upon thoracoscopic NIR evaluation, the median tumor fluorescence intensity was noted to be 91.0 AU (IQR, 83-105 AU) (Figure 1e), which was higher than within surrounding benign pulmonary parenchyma which measured 28.5 AU (IQR, 23-45 AU) (Figure 1f); p<0.001 (Figure 1g). The median *in situ* TBR of nodules displaying *in situ* fluorescence was 2.4 (IQR, 2.2-3.7). The median size of the 7 *in situ* fluorescent tumors was 1.7cm (range, 1.1-4.6 cm). Based on preoperative imaging, each of these tumors were sub-pleural and located within 1.2cm of the lung surface.

**Figure 2:**
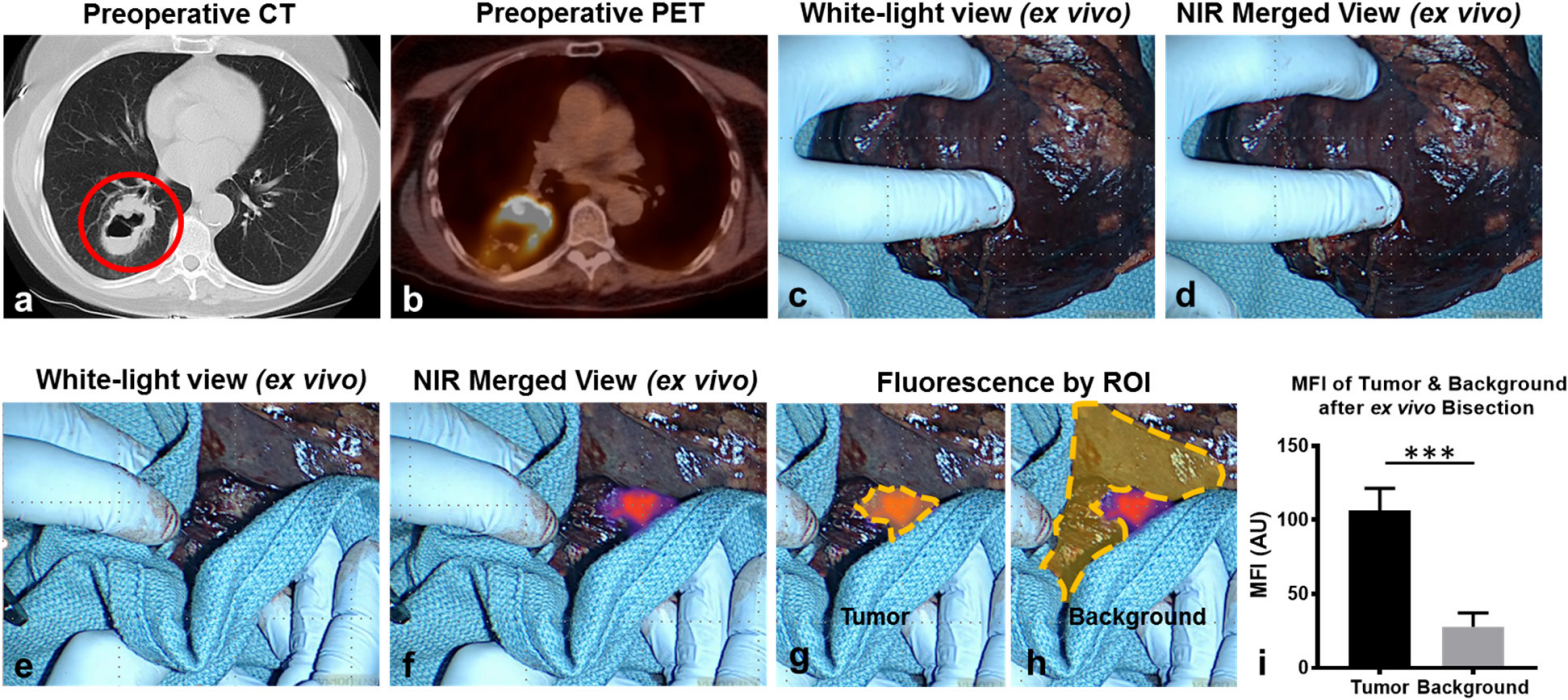
SCCs display strong fluorescence upon *ex vivo* tumor bisection Representative example: Subject 1 presented with a 5.2cm right lower lobe nodule **(a)** that displayed an SUV of 13.1 **(b)**. During *in situ* and *ex vivo* analysis, no parenchymal changes were appreciated during white light evaluation **(c)** nor during NIR molecular imaging **(d)**. Upon *ex vivo* tumor bisection, the tumor was visualized both by white light inspection **(e)** and by NIR molecular imaging **(f)**. Fluorescent intensity was quantified for ROIs corresponding to the tumor **(g)** and benign pulmonary parenchyma (background) **(h)**. Mean fluorescent intensities were compared for SCCs (n=9) displaying *ex vivo* fluorescence during back-table inspection **(i)**. Red circle-pulmonary SCC; yellow gates-ROIs measured by fluorescent analysis; ^***^p<0.001.

After resection and direct tumor exposure during *ex vivo* tumor bisection, two SCCs which were non-fluorescent by thoracoscopic assessment displayed obvious e*x vivo* fluorescence upon tumor bisection (Subject 1 and Subject 10) (Figure 2a-2f; Supplementary Video 2). All (7 of 7) SCCs exhibiting *in situ* signal also displayed fluorescence upon exoscopic FR-IMI. In total, 9 of 13 (sensitivity 69.2%; false negative rate of 31.8%) evaluated SCCs evaluated (in 9 of 12 subjects) displayed strong signal during FR-IMI. We noted uniform tumor-associated fluorescence throughout the tumor surface with a clear demarcation noted between the tumor margin and benign surrounding lung parenchyma (Figure 2f). Median fluorescence of these 9 SCCs was nearly 4-fold higher than signal of surrounding benign lung (103 vs 29 AU; p<0.001) (Figure 2g-2i). The median TBR of those SCCs displaying *ex vivo* fluorescence upon bisection was 3.9 (IQR, 3.5-4.2); which was higher than TBRs observed during *in situ* fluorescent evaluation; *p*=0.02. Of note, lesions displaying only signal upon *ex vivo* evaluation were similar in size to those displaying *in situ* (median 4.1cm vs 1.7cm; p=0.18); however, were deeper than those also displaying *in situ* signal (median 3.0cm vs. 0.2cm; p<0.01).

### Folate receptor-targeted OTL38 accumulation within SCCs

**Figure 3:**
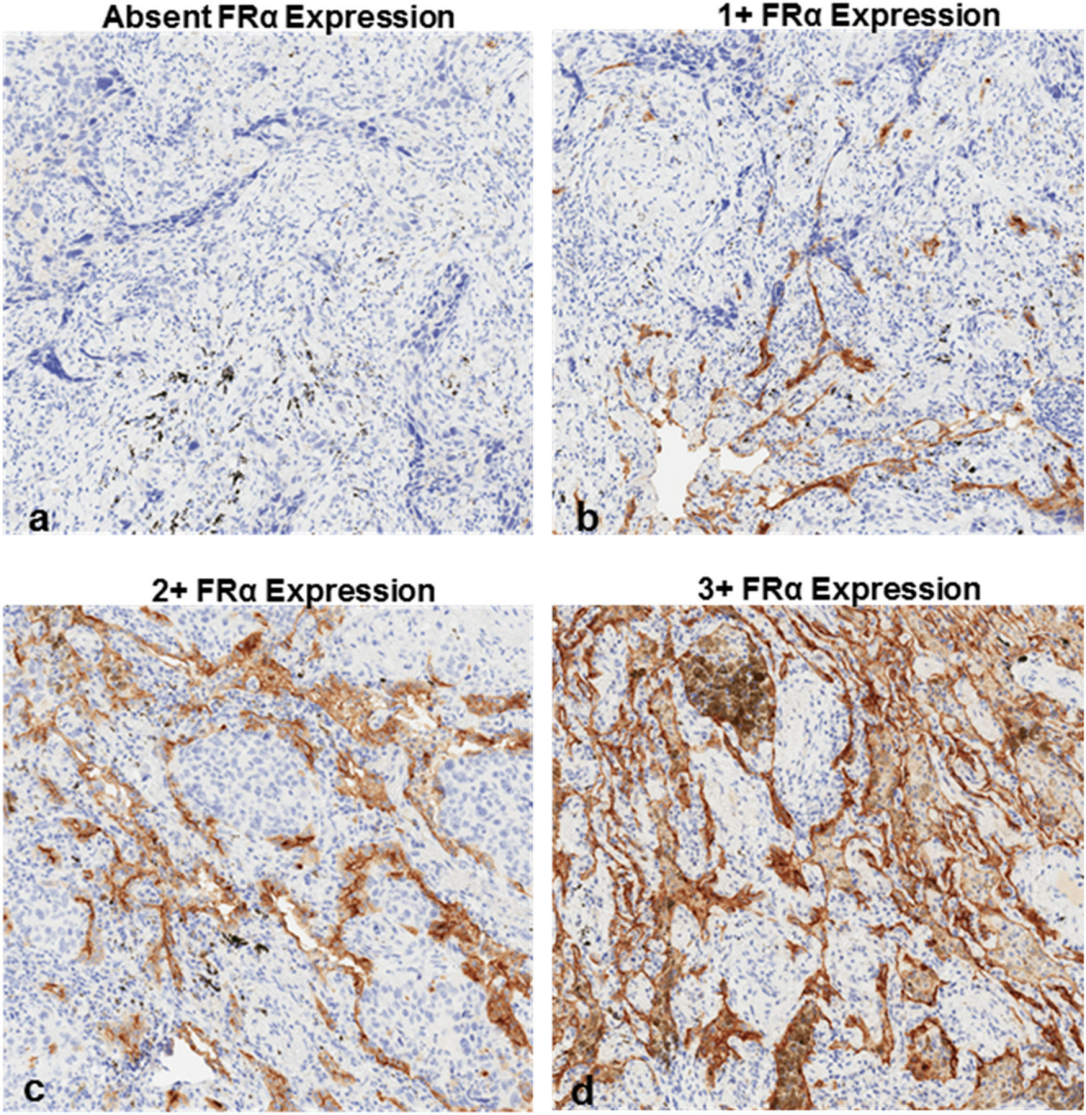
FRα expression patterns in SCCs as determined by immunostaining **(a)** absent FRα expression, **(b)** 1+ FRα expression, **(c)** 2+ FRα expression and **(d)** 3+ FRα expression. 2+ and 3+ staining patterns were considered overexpression.

Of those SCCs demonstrating fluorescence upon FR-IMI, each demonstrated FRα expression (1+, 2+ or 3+) upon immunostaining (representative examples of 0, 1+, 2+ and 3+ staining provided in Figure 3). Three (33.3%) FRα-expressing tumors were found to have predominantly 1+ staining, 4 (44.4%) had predominantly 2+ staining patterns, and 2 (22.2%) had predominantly 3+ staining. In each FRα-positive specimen, heterogenous expression patterns were appreciated with each specimen displaying areas of 0, 1+, 2+ and 3+ staining. Of note, FRα-expression was absent in the three non-fluorescent SCCs submitted for histopathologic review.

**Figure 4:**
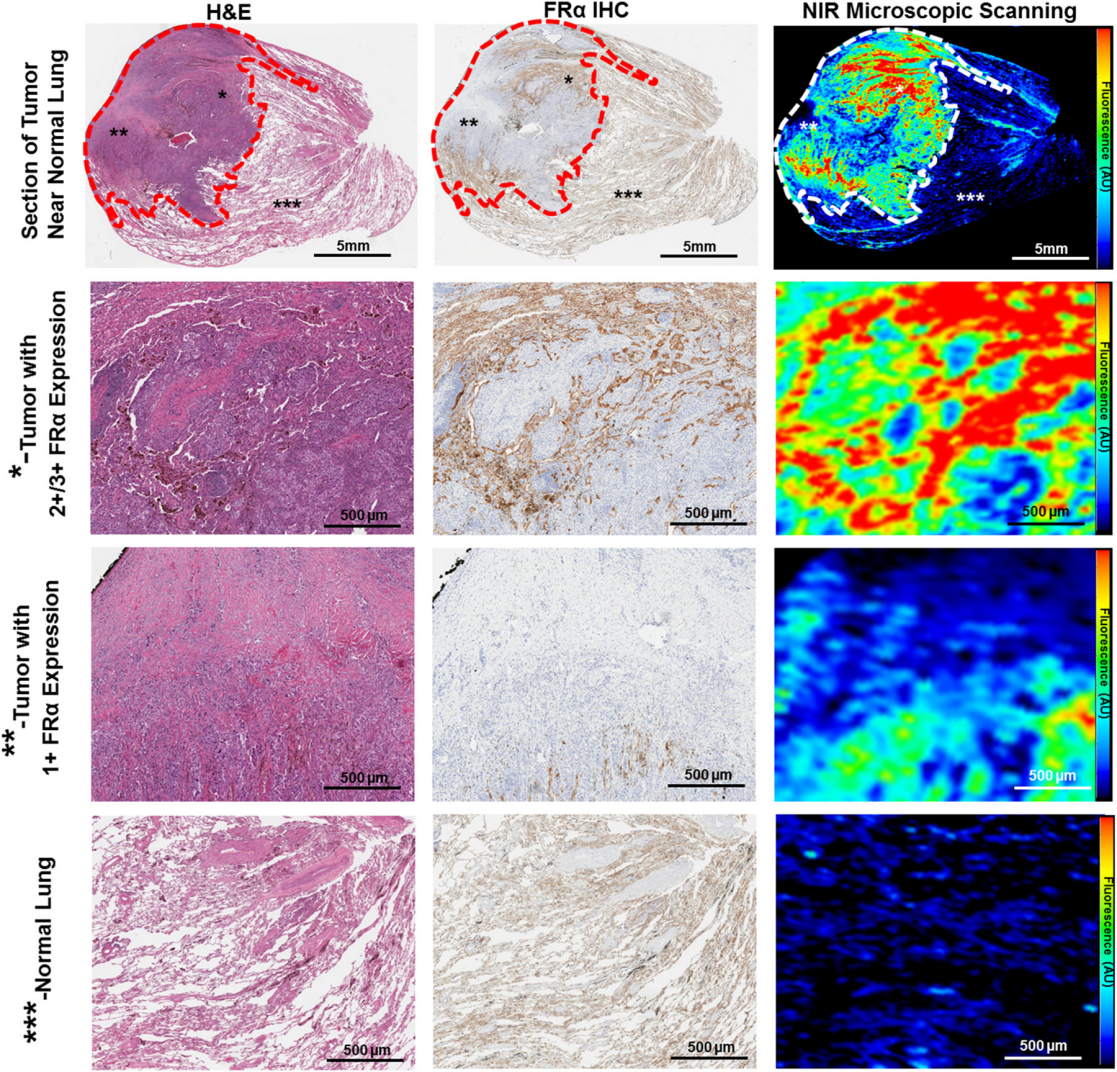
OTL38 accumulation within SCC is FRα-dependent Each resected SCC underwent microscopic fluorescent scanning and correlative molecular analysis. Representative comprehensive analysis of resected SCC (Subject 9). Whole-section images were obtained and evaluated using H&E staining, FRα IHC and a NIR microscopic scanning (top row). The tumor (gated in in dash marks) demonstrated strong fluorescence, particularly in areas of FRα expression. We noted increased fluorescence in areas of FRα overexpression (^*^- second row), and moderate levels in those areas with 1+ FRα staining (^**^-third row). Normal pulmonary parenchyma displayed negligible fluorescence (^***^-bottom row). ^*^ - Area of tumor with FRα overexpression (2+/3+ Staining), ^**^ - Area of tumor with low FRα expression (1+ or absent), ^***^ - normal lung parenchyma.

To better characterize patterns of OTL38 accumulation within SCC and confirm accumulation in FRα-expressing tumors areas, all fluorescent SCC underwent microscopic tumor mapping using a NIR scanning system and molecular correlative analysis using a combination of H&E staining and FRα immunohistochemistry (representative analysis provided in Figure 4). After noting tumor margins by of H&E and IHC, quantitative fluorescent analysis confirmed elevated mean fluorescence intensities within tumors as compared to normal benign pulmonary parenchyma (versus 98.1 AU vs 20.3; p<0.001) (Figure 4). Within tumors, we noted significant increases in fluorescent intensity in those areas with FRα-overexpression (2+ or 3+ staining) versus those areas with lower expression patterns (140.6 AU vs 57.2 AU; p<0.001).

### FR-IMI improves localization of T1a squamous cell carcinomas

**Figure 5:**
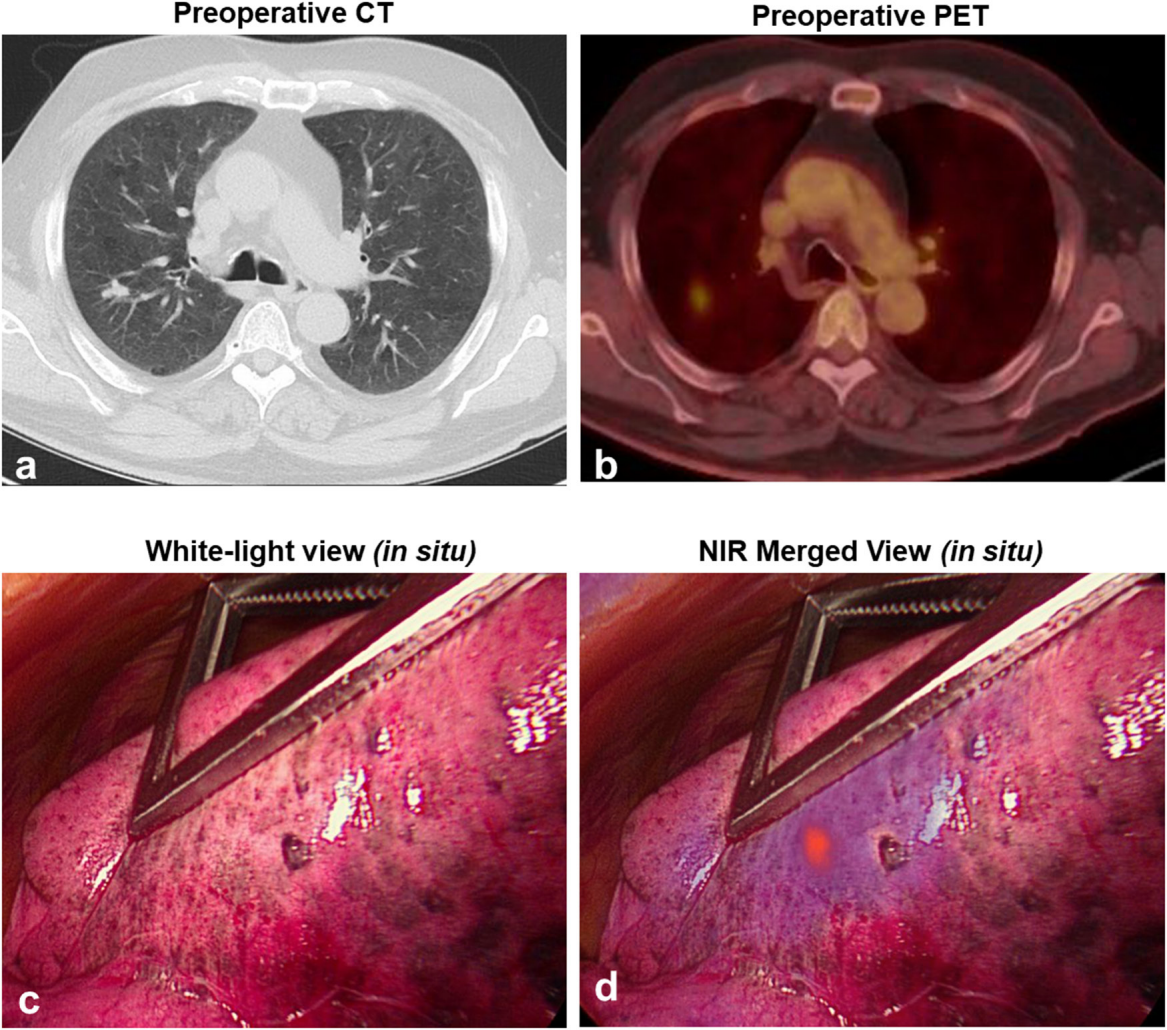
Representative example displaying FR-IMI localization of a small SCCs Subject 9 presented with a metabolically active 1.1cm nodule of the right upper lobe as determined by preoperative CT **(a)** and PET **(b)**. Intraoperatively, the nodule could not be localized using standard visualization techniques **(c)**; however, upon FR-IMI that nodule highly visible **(d)**.

After confirming FRα-dependent accumulation of OTL38 within SCC, we reviewed how FR-IMI impacted clinical care. In 3 of 12 subjects, utilization of FR-IMI impacted surgical care by allowing for intraoperative localization of T1a lesions (measuring less than 2.0cm). For example, Subject 9 presented with a ^18^FDG-avid, 1.1cm right upper lobe nodule (Figure 5a, 5b). This patient was taken to the operating room for a VATS wedge resection. During standard thoracoscopic localization (white light and finger palpation), the nodule could not be localized through the pleural surface (Figure 5c). However, after implementation of *in situ* NIR molecular imaging, the nodule was readily identified (Figure 5d). Using the real-time fluorescent feedback, the lesion was successfully resected and adequate margins were confirmed by exoscopic fluorescent evaluation. Fluorescent results were confirmed by both frozen section and final pathologic evaluation. Without the addition of FR-IMI, the operating surgeon would have otherwise converted from VATS to thoracotomy to accomplish localization and resection. Similarly, in Subject 5 and Subject 12 the addition of FR-IMI allowed for the resection of T1a nodules to continue in a minimally invasive fashion.

### Histopathologic and clinical variables predicting *in-situ* fluorescence of SSCs during FR-IMI

To maximize utility of FR-IMI for localization, *in situ* intraoperative fluorescence is paramount. To determine which patient and tumor variables predict *in situ* signal upon FR-IMI; several clinical, radiographic and histopathologic variables were assessed: depth from pleural surface on preoperative CT, time from infusion to imaging, preoperative SUV, tumor location, SCC size, amount of drug given, subject age and final subject gender (Table 3). Of the 9 tumors which accumulated OTL38, we determined that only depth from pleural surface impacted *in situ* fluorescence, with deeper nodules associated with less fluorescence (p=0.005). In fact, among the 12 subjects analyzed, only those with SCCs within 1.2cm of the pleural surface displayed identifiable signal during *in situ* FR-IMI. Other trends predicting increased *in situ* TBR included longer time to imaging, however, this did not reach significance (p=0.06). No other examined variable predicted *in situ* tumor fluorescence.

**Table 3:** Variables predicting *in situ* fluorescence

Characteristics of fluorescent SCCs (n=9)	Coefficient	95% CI	p-value
**Depth of Nodule (cm)**	-0.61	-0.96 to -0.25	**0.005**
**Time to Imaging (hours)**	0.39	-0.01 to 0.80	0.06
**Standard Uptake Value by PET**	0.29	-0.08 to 0.23	0.29
**Nodule Location (RUL, RML, RLL, LLL, vs LUL)**	0.20	-0.48 to 0.89	0.50
**Nodule size (cm)**	-0.15	-1.59 to 1.28	0.80
**Amount of drug given (mg)**	0.71	-1.15 to 2.57	0.81
**Age (years)**	0.02	-0.18 to 0.23	0.81
**Gender (M vs F)**	-0.06	-1.77 to 1.64	0.93

RUL-Right Upper Lobe; RML-Right Middle Low; RLL-Right Lower Lobe; LUL-Left Upper Lobe; LLL-Left Lower Lobe; M-Male; F-Female.

## DISCUSSION

More than 80,000 patients undergo resection for NCSLC in the United States each year [1]. Localization of pulmonary nodules, particularly during minimally invasive pulmonary resection, can be challenging. Our group has previously demonstrated that FR-IMI with OTL38 can be a safe and reliable intraoperative adjunct to improve localization of pulmonary adenocarcinomas. In this study, we expand upon our previous experiences, and demonstrate FR-IMI with OTL38 can also be a useful surgical adjunct for patients undergoing pulmonary resection of pulmonary SCCs. We demonstrate that OTL38 accumulates in nearly 70% of SCCs by exhibiting FRα-targeted mechanisms. Reproducible fluorescent signal during FR-IMI improved intraoperative localization of small, subpleural nodules during minimally invasive resection.

OTL38 was developed in efforts to incorporate advantages of several alternative imaging agents currently under Phase I/II evaluation. More specifically, OTL38 incorporates highly specific FRα-targeted binding as previously observed with EC17 (folate-FITC) [18]. Unlike EC17, folate is linked to S0456 (a commercially available NIR dye) in OTL38 [23]. NIR imaging agents offer several advantages over visual-range agents including minimal autofluorescence and improved depth of tissue penetration [17]. Given an abundance of data describing FRα expression within 70-80% of pulmonary adenocarcinomas [24], we chose to initiate first-in-human studies using this histology as a study model. In these early trials involving more than 70 patients; FR-IMI with OTL38 was noted to be safe, highly sensitive in localizing small pulmonary adenocarcinomas (as small as 2-3mm), and capable identifying occult malignancy in 12% of subjects enrolled [3, 13].

In light of high reproducibility and excellent sensitivity of FR-IMI within pulmonary adenocarcinomas, we chose to explore FR-IMI with OTL38 in the second most common NSCLC histology, SCCs. SCCs account for nearly 30% of all lung cancers, and nearly 35% of all NSCLC histologies. Unlike pulmonary adenocarcinomas, previous data describing FRα expression patterns in SCCs was unclear and ranged from 10% to nearly 60% [20, 21]. In addition to inconsistent expression values, previous reports have been limited due to a small sample size, sampling error introduced by microarray analysis, and poor generalizability to surgical patients. Because of these limitations we sought to execute a feasibility study aimed at exploring FR-IMI in patients with resectable pulmonary SCC.

In this study, we were encouraged to that find 9 of 12 subjects successfully underwent FR-IMI with OTL38. In addition to reproducibility, we noted that FR-IMI provided subcentimeter sensitivity and an effective adjunct to minimally invasive pulmonary resection. Upon fluorescent and correlative histopathologic assessment, we confirm that OTL38 accumulation in SCCs was indeed targeted to FRα expressing areas within tumor. In total, 69% of SCCs displayed FRα expression. We point out that higher rates of FRα expression in our cohort as compared to previous studies may be explained by several factors, perhaps most notably involves our histologic evaluation approach which utilized whole sections as compared to tissue microarrays in previous studies. Furthermore, we observed significant intratumoral heterogeneity in FRα expression as compared to our prior experiences involving pulmonary adenocarcinomas. Nevertheless, these results support our previous findings insomuch tumors display macroscopic fluorescence when as little as 10% cells display the FRα [3, 13, 25]. These results provide an encouraging platform for future studies that can better clarify these trends in pulmonary squamous cell carcinomas.

Similar to previous studies involving FR-IMI and alternative NIR optical contrast agents, we found significant limitations of with tumors deeper than 1.2cm. This mirrors our experiences with pulmonary adenocarcinomas where *in situ* nodule localization was ineffective for lesions deeper than 2.0cm [3, 13]. At this time a number of additional techniques, such as photoacoustic imaging and NIR-endoscopy, are in development. These approaches have proven successful for metastatic melanoma [26] and head and neck malignancies [27], respectively.

Despite these limitations, molecular imaging offers several advantages over alternative localization approaches currently available. First, from a purely logistical perspective, molecular imaging requires no additional procedures which contrasts with CT-guided percutaneous wire localization, fluoroscopy, fiducial marking, and tumor tattooing [28]. In comparison, during FR-IMI OTL38 is delivered just hours prior to resection in the preoperative holding area. Secondly, FR-IMI for lung cancer has demonstrated an excellent safety profile with only grade I toxicities noted [3, 13]. We note a good safety profile in this trial, with no adverse events being observed in this study cohort. This provides important morbidity advantages over previously mentioned localization techniques [29]. Finally, systemic drug delivery involved in IMI provides an opportunity for detection synchronous disease and lymph nodes evaluation as previously noted in our experiences with FR-IMI in pulmonary adenocarcinomas and other groups investigating antibody-based approaches [3, 30].

Despite limitations, we are optimistic about the role of FR-IMI for patients with pulmonary SCCs and view these results as a template for additional studies which can help better define how FR-IMI can improve upon standard treatment approaches for NSCLC patients. More specifically, we are currently exploring FR-IMI with OTL38 as a tool to improve localization of ill-defined pulmonary ground-glass opacities (NCT02769156). In addition to this registration trial, we are embarking on a multi-center Phase II trial (NCT02872701) involving FR-IMI OTL38 which will mark the United States’ first trial involving targeted IMI. These additional investigations will provide a better understanding of how FR-IMI can impact care for patients undergoing minimally invasive pulmonary resection for NSCLC.

## MATERIALS AND METHODS

### Study design

This clinical trial was approved by the University of Pennsylvania’s Institutional Review Board. The primary objectives of this study were to determine if OTL38 accumulates within SCCs and to assess *in vivo* and *ex vivo* tumor fluorescence. Secondary objectives included the determination of fluorescent dependence on patient and tumor variables including FRα expression.

Twelve subjects provided informed consent and were enrolled between July 2015 and January 2017. Sample size and study design were based on consensus guidelines provided by the World Molecular Imaging Society [31]. All subjects presented with solitary pulmonary nodules that were histologically confirmed to be SCCs. All identified subjects underwent preoperative high-resolution CT scanning which was reviewed by a thoracic radiologist to confirm the presence pulmonary nodule and identify other suspicious nodules. Each subject also underwent preoperative ^18^FDG-positron emission tomography (PET). For imaging studies obtained outside the University of Pennsylvania Health System (UPHS), at least one UPHS thoracic radiologist reviewed preoperative imaging to confirm the presence of pulmonary nodules, rule-out synchronous disease, and confirm findings of ^18^FDG-positron emission tomography. Based on limitations in depth of penetration recognized in our prior experience with NIR imaging, we selected subjects SCCs within 4cm of the visceral pleural surface on preoperative imaging [13]. A Transparent Reporting of Evaluations with Nonrandomized Designs (TREND) flow diagram with complete inclusion and exclusion criteria is provided (Supplementary Figure 1).

All subjects were scheduled for minimally invasive pulmonary resection (video-assisted thoracic surgery—VATS). In the preoperative holding area, study participants received 0.025 mg/kg of OTL38 by intravenous infusion 3-6 hours prior to resection as previously described [13]. During VATS, surgeons first utilized standard thoracoscopic visualization (white-light thoracoscopy) and finger palpation to identify known tumors. After identification of preoperatively identified nodules, molecular imaging was used to confirm lesion fluorescence. If the preoperatively identified nodule was unidentifiable by white-light thoracoscopy or finger palpation, localization using fluorescence guidance was attempted. If additional fluorescent lesions were identified, they were wedge resected. All specimens were then imaged *ex vivo* prior to submitting for histopathologic examination by a pulmonary pathologist.

### Study drug

OTL38 (C_61_H_63_N_9_Na_4_O_17_S_4_; molecular weight: 1414.42 Da) is composed of a folate analogue conjugated to an NIR fluorescent dye as previously described [3]. OTL38 maximally excites at a wavelength of 774-776 nm and has a peak emission of 794-796 nm [3, 9]. OTL38 (>96% purity) was obtained from On Target Laboratories (West Lafayette, IN, USA). All subjects received 0.025 mg/kg of intravenous OTL38 through a peripheral vein 3 to 6 hours prior to resection. OTL38 dosage and time to imaging was based on previous human [3, 9] and canine pharmacokinetic data [32].

### Imaging device

*In situ*, real-time fluorescent imaging was performed using an Iridium® system optimized for detection of OTL38 (Visionsense Corp, Philadelphia, PA) as previously described [3]. The Iridium® is a high definition, dual band (white light and NIR) camera system capable of emitting and detection light in the NIR spectrum. An excitation source with a wavelength of 785 nm was utilized, and fluorescence was detected using a bandpass filter selective to light ranging from 800 to 835 nm. During VATS, the Iridium® was equipped with a 5mm, 0-degree thoracoscope. During *in situ* imaging during VATS, the distance was normalized using the Iridium®’s “distance lock” feature. For *ex vivo* back table evaluation of specimens, a free-standing exoscope was utilized. By exoscopic evaluation, imaging was performed at a distance of 15 inches. Of note, during both *in situ* and *ex vivo* imaging, the “autogain” function was utilized, and power was maintained at the default setting.

### Fluorescent and histopathologic assessment of specimen

Excised specimens were formalin fixed and paraffin embedded. Sequential 5μm sections were obtained and underwent comprehensive histopathologic and fluorescent analysis by a board-certified thoracic pathologist. Sections were stained using standard hematoxylin/eosin (H&E) staining. Immunohistochemical (IHC) staining for FRα was performed using a monoclonal antibody, 26B3.F2 (Morphotek Inc., PA) as previously described [13]. Once stained, a certified pulmonary pathologist manually scored specimen using an established scoring system ranging from 0 to 3+ as previously described [13]. Briefly, a score of 0 corresponded with absence of staining; 1+ equaled faint staining on luminal borders; 2+ equaled moderate staining on apical and sometimes lateral borders and 3+ indicated strong circumferential staining. The tumor was considered positive when more than 10% of malignant cells were positively stained. Lastly, in order to understand OTL38 accumulation patterns at a microscopic level, an additional unstained 5μm section was evaluated using a NIR microscopic scanner (*Odyssey*, LiCor, Lincoln, NE).

### Statistical analysis

*Post hoc* image analysis was performed to quantify the amount of fluorescence using region of interest (ROI) software within ImageJ (free software program available through the National Institute of Health; http://rsb.info.nih.gov/ij). A background fluorescence level was similarly obtained, and tumor-to-background (TBR) was calculated for all identified lesions. A TBR was utilized to control for extrinsic variables including ambient operating room signal, natural fluorophores, and systemic patient variables. Data are presented as median (IQR) unless otherwise noted. Given the small sample size, differences between two groups were compared by the Mann-Whitney test. Given the exploratory nature of this study, multiple prediction models were made using linear regression to assess patient and histopathologic variables that related to tumor fluorescence. All comparisons were made use Stata Statistical Software: Release 14 (College Station, TX: StataCorp LP). A p-value of 0.05 or less was considered statistically significant.

## SUPPLEMENTARY MATERIALS FIGURE AND VIDEOS







## Abbreviations

AE: adverse event
BMP: basic metabolic panel
CBC: complete blood count
CT: computed tomography
CTCAE: Common Terminology Criteria for Adverse Events
FRα: folate receptor alpha
H&E: hematoxylin and eosin
ICG: indocyanine green
IHC: immunohistochemistry
IMI: intraoperative molecular imaging
IQR: interquartile range
LFTs: liver function tests
NaCl: sodium chloride
NIR: near infrared
NSCLC: non: small cell lung cancer
PET: positron emission tomography
ROI: region of interest
SUV: standardized uptake value
TBR: tumor to background fluorescence ratio
VATS: video assisted thoracic surgery
