# Supplementary material for: An open label trial of folate receptor-targeted intraoperative molecular imaging to localize pulmonary squamous cell carcinomas
Source: Oncotarget. 2018 Feb 5;9(17):13517–29. doi: 10.18632/oncotarget.24399 (PMC5862595; doi:10.18632/oncotarget.24399)
Supplement: Supplementary file 1 [file oncotarget-09-13517-s001.pdf]

An open label trial of folate receptor-targeted intraoperative molecular imaging to localize pulmonary squamous cell carcinomas

SUPPLEMENTARY MATERIALS

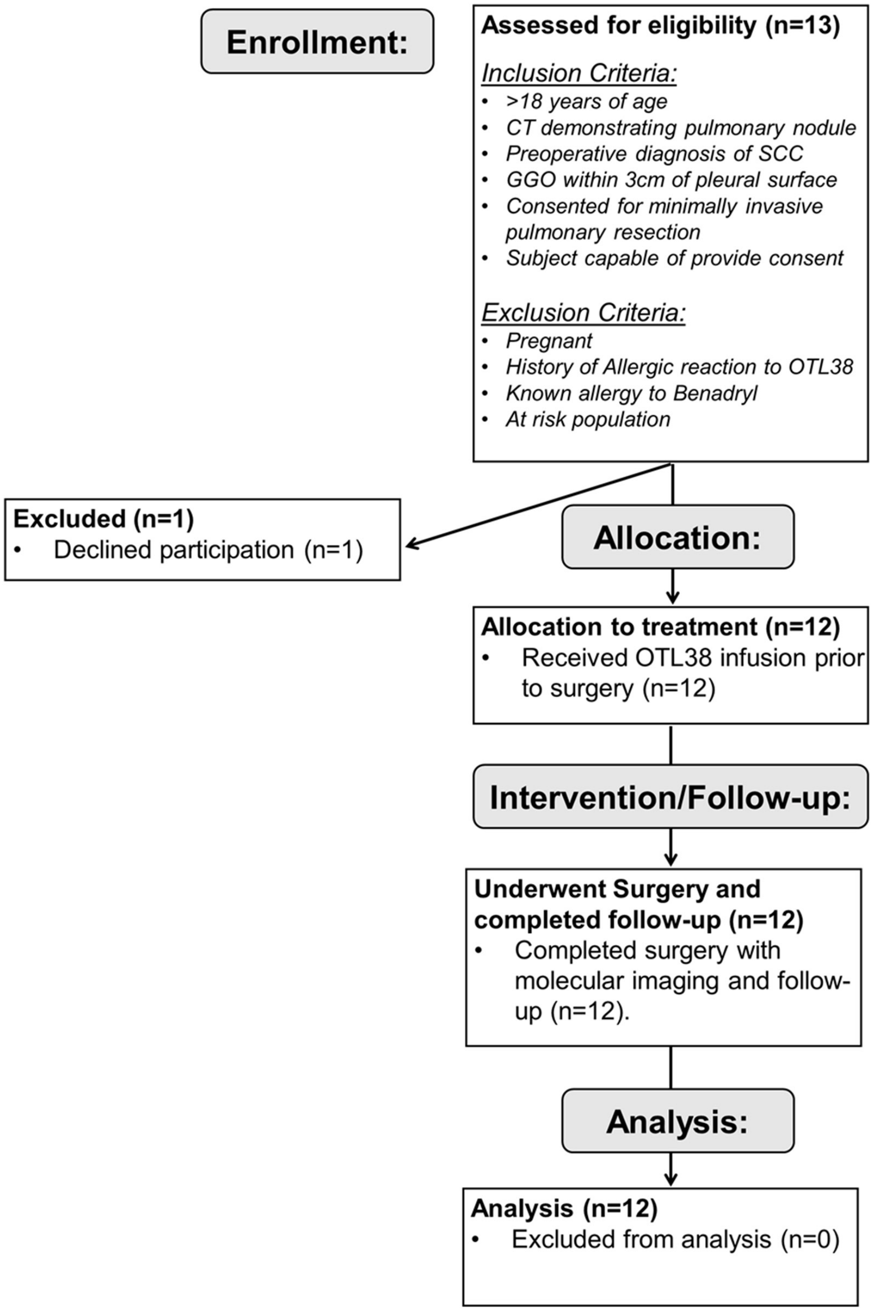

Supplementary Figure 1: Transparent Reporting of Evaluations with Nonrandomized Designs (TREND) Flow Diagram of Trial.

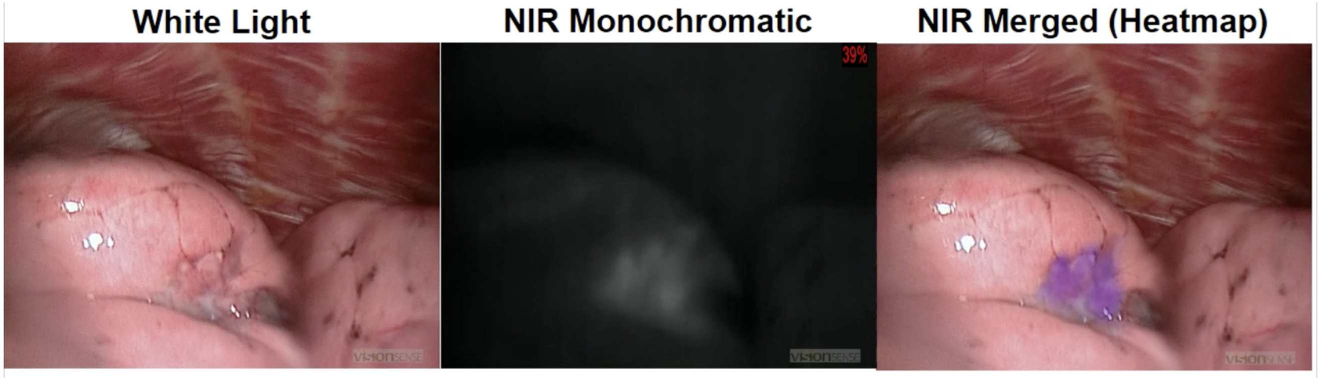

Supplementary Video 1: *In situ* fluorescence of SCC during FR-IMI with OTL38. Subject 3 presented with a 1.7cm left upper lobe nodule which displayed strong NIR signal during *in situ* fluorescent imaging.

See Supplementary Video 1

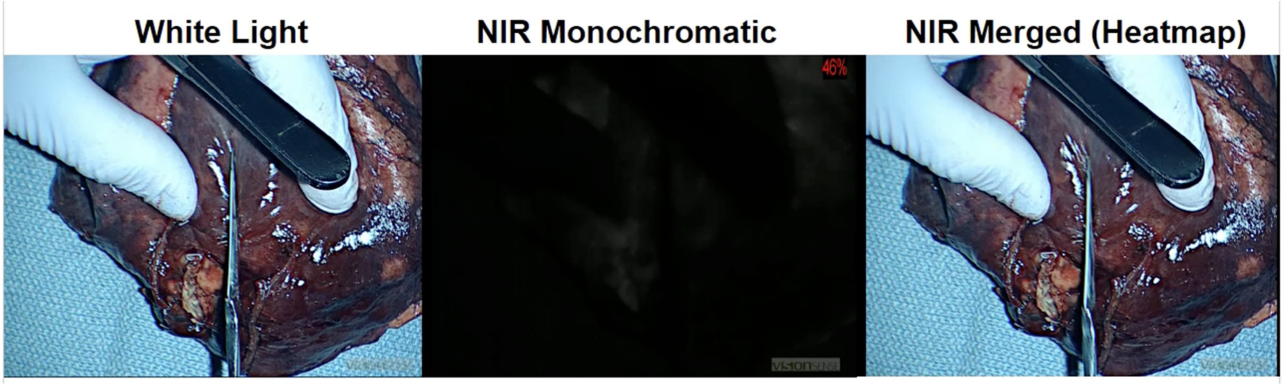

Supplementary Video 2: SCC displaying fluorescence upon *ex vivo* tumor bisection. Subject 1 presented with a 5.2cm right lower lobe nodule which displayed fluorescence only after tumor bisection.

See Supplementary Video 2
